# Analysis and control of information diffusion dictated by user interest in generalized networks

**DOI:** 10.1186/s40649-015-0025-4

**Published:** 2015-12-02

**Authors:** Eleni Stai, Vasileios Karyotis, Symeon Papavassiliou

**Affiliations:** School of Electrical and Computer Engineering, National Technical University of Athens (NTUA), 15780 Zografou, Athens, Greece

**Keywords:** User interests, Information diffusion, Generalized networks, SIS epidemic model, Time-varying interests, Optimal control, Pontryagin’s Maximum Principle, Hamilton–Jacobi–Bellman equation

## Abstract

The diffusion of useful information in generalized networks, such as those consisting of wireless physical substrates and social network overlays is very important for theoretical and practical applications. Contrary to previous works, we focus on the impact of user interest and its features (e.g., interest periodicity) on the dynamics and control of diffusion of useful information within such complex wireless-social systems. By considering the impact of temporal and topical variations of users interests, e.g., seasonal periodicity of interest in summer vacation advertisements which spread more effectively during Spring–Summer months, we develop an epidemic-based mathematical framework for modeling and analyzing such information dissemination processes and use three indicative operational scenarios to demonstrate the solutions and results that can be obtained by the corresponding differential equation-based formalism. We then develop an optimal control framework subject to the above information diffusion modeling that allows controlling the trade-off between information propagation efficiency and the associated cost, by considering and leveraging on the impact that user interests have on the diffusion processes. By analysis and extensive simulations, significant outcomes are obtained on the impact of each network layer and the associated interest parameters on the dynamics of useful information diffusion. Furthermore, several behavioral properties of the optimal control of the useful information diffusion with respect to the number of infected/informed nodes and the evolving user interest are shown through analysis and verified via simulations. Specifically, a key finding is that low interest-related diffusion can be aided by utilizing proper optimal controls. Our work in this paper paves the way towards this user-centered information diffusion framework.

## Introduction

Analyzing and controlling information diffusion in complex networks is of high research and practical interest nowadays. “Information” may appear in diverse forms, useful or malicious, each with different diffusion dynamics and demanding different types of control. Malicious information, e.g., a dangerous computer virus, might have catastrophic outcomes calling for suppressive control, while marketing advertisements can be exploited for maximizing online revenues and may be enhanced by an amplifying type of control.

To better facilitate the increased needs for effective information exchange, continuing technological advances in wireless and wired communications and the development of online social networks have given rise to “generalized” network systems. The latter consist of a physical layer, i.e., a wireless medium, and a social overlay, where social encounters develop, forming combined cyber-physical, e.g., social-wireless networks, referred to as generalized networks [1]. According to [2], generalized networks, even when consisting of a physical (e.g., wireless multihop) and only one social network can significantly improve information spread. In this paper, we focus on social-wireless types of generalized networks, while other types may be straightforwardly considered.

Motivated by the above observations on networks and information proliferation, in this paper, we focus on the diffusion dynamics and control of useful information in generalized networks. Various relevant works on the topic exist in the literature ("Related work and contributions"). However, albeit, they bear a specific drawback by not accounting for the evolution of user interest on the information diffused. Typically, humans interact with each other and exchange content on the basis of features such as “topics of information”. In particular, during an encounter, humans may not care for information that is out of their interest range at that particular time, thus not participating in the diffusion of the corresponding topic. Therefore, communicating information is highly affected by user interests and their temporal evolution, since not every contact does necessarily imply information transfer for all the topics under diffusion. It rather depends both on human preferences and their interconnections (physical and social topology).

Several real-world examples indicate the dependence of information diffusion dynamics on the temporal and topical variation of user interests [3–5]. For instance, advertisements on summer vacations are expected to have a more successful spread outcome during the Spring and Summer months, while being hobbled during Fall and Winter months, highlighting an emergent seasonal periodicity with respect to user interests. Secondly, news on a soccer match might not be well spread within the members of a dance group, while they are expected to be quickly spread within the members of a soccer club. The first of the above cannot be expressed by the current models of information diffusion which do not segregate the diffusion success rate with respect to seasonal dependence, while the second case implies a non-homogeneous information rate across populations with different characteristics. As a result, for a realistic inclusion of users’ interests in the information diffusion model, the interests should be considered time varying, e.g., reflecting the evolving seasonal behavior of human beings [6]. The second example further implies the need of explicitly taking into account the subject of users’ interests, when designing information diffusion models.

Thus, in this paper we introduce and develop for the first time an information diffusion modeling framework that takes into account both user interests’ differentiation and their possible temporal variability (e.g., periodicity). Furthermore, we provide an optimal control framework on top of the information diffusion model that allows for trading-off diffusion efficiency with the associated cost, leveraging on the impact that user interest has on information dissemination dynamics. To the best of our knowledge, there is limited literature in the field of optimal control over diffusion dynamics described by epidemic modeling with time-evolving parameters [6, 7]. Incorporating control, will benefit information spreading, particularly when there is limited interest on the useful information being diffused, in which case, it can be mapped to, e.g., advertising campaigns or other incentives provided to users in an optimized way with respect to cost. An example of explicit control is the provision of incentives to users, e.g., in the form of competition, rewards, reputation, etc., to participate in information propagation when their interest itself in the propagated topic is limited, decreasing in this way the probability that information propagation on a specific subject deceases fast enough. Significant outcomes are provided on the impact of each topological layer (social or wireless) and the associated interest parameters on the dynamics and control of information diffusion over complex social-wireless topologies, via analysis, numerical evaluations and simulations of relevant scenarios. Furthermore, the properties/behavior of the optimal controls on information diffusion are extensively studied.

The rest of the paper is organized as follows. "Related work and contributions" describes related literature and positions our work within the existing relevant literature, while "System model, notation and assumptions" presents the employed system model. "Information diffusion modeling and analysis without control" analyzes the proposed information diffusion model and the examined application scenarios. In the sequel, "Optimal control framework for information diffusion" introduces the information diffusion optimal control framework, while "Simulation and numerical results without applying control" and "Simulation results, numerical results and discussion in controlled information diffusion" present and thoroughly discuss the performed simulation results and numerical evaluations without and with control, respectively. Finally, "Conclusions" concludes the paper.

## Related work and contributions

Due to the importance of information nowadays, studying the properties of its diffusion along with the possibility of control has attracted considerable interest. In this paper, we focus on two important facets of information diffusion, namely the dynamics of information spreading and its optimal control.

Regarding the dynamics of information diffusion, the earliest and most frequently encountered approaches were inspired by epidemiological models [8, 9]. Some of the most recent ones are [1, 10–13], while more can be found in the references therein. More specifically, both stochastic and deterministic epidemic models exist for information propagation [7, 14], where the nodes having received the information are denoted as “infected”. Stochastic epidemic models treat information propagation as a discrete time process (Discrete Time Markov Chain) [7, 14] being more suitable for small-scale systems whereas deterministic epidemic models assume continuous processes relying on the law of large numbers and applying differential equations or inclusions [15, 16], thus being more suitable for large-scale systems. In this paper, we will apply a deterministic epidemic model. Most of deterministic models consider the evolution of the cumulative system state/number of infected individuals (macroscopic modeling), denoted as “population dynamics”, assuming homogeneous infection rates for all population members. On the other hand, the deterministic “network” models study the state of each individual separately and also segregate infection rates between different pairs of individuals [7]. However, system state transitions (i.e., population dynamics) depend on the state transition models developed for each individual, e.g., susceptible–infected–susceptible (SIS), etc. [7, 8]. Contrary to deterministic network models, a typical assumption when considering population dynamics is that of homogeneous mixing, where contact patterns between individuals are considered highly homogeneous [17]. Both types of models, stochastic and deterministic, account for the endogenous (transition that takes place owe to internal individual operation, e.g., recovery transition) and exogenous (transitions dictated by external factors, e.g., infection transition) transition rates expressing the topological and operational, endogenous or exogenous, factors that affect the evolution of the system [7].

Information dissemination epidemic models have been developed for different network topologies, e.g., wireless networks [18], social networks [8] and multiple social networks [3], and generalized networks [1]. More specifically, epidemic models, e.g., SIS, susceptible–infected–removed (SIR) and susceptible–infected (SI) [6, 8] have been adopted and adapted over diverse network topologies to describe the spreading of useful or malicious information. In this work, we mainly focus on the diffusion of useful information over generalized networks based on the SIS epidemic model. Our model lies between the frameworks of population dynamics and network models, since we study system state transitions while considering neighborhood relations in a node-degree sense.

Furthermore, to the best of our knowledge, the impact of user interests and their temporal variability analyzed in this work have been considered in the literature in a limited degree, e.g., [3, 4, 19]. In [3], the authors aim at finding the minimum number of seed users who can spread the information to all users interested in the specific topic over multiple online social networks (where some users belong in more than one online social networks simultaneously). The work in [19] studies the role of information diffusion to the evolution of the network topology considering the link formation process with respect to sources/retransmitters of the information, based on users’ preferences. User interests in the information topics being propagated are also inferred and considered in [4] to detect active links in the diffusion of a given message over the network. Generally, most of the previous works, except from taking into account contact-related and topological factors affecting information diffusion dynamics [1, 13], they occasionally regard static users’ interests [3, 4], but not the user interest temporal variability. In a closer spirit to our approach, [6] studies the spatio-temporal dynamics of information diffusion via partial differential equations, while incorporating time decreasing users’ interest on the propagated messages. A similar study is performed for the case of malware dissemination over the Internet in [15]. Specifically, in [15], the infection rate decreases with time while this time dependence is shown via experimentation to model better the Code Red worm propagation. The reason for such decreasing infection rate is that worm spread over the Internet can be slowed down by countermeasures employed by users and congestion points arising over Internet. As in [15], in our approach, the introduction of the time evolving users’ interests in the information propagation decisions along with the consideration of a network substrate abolishes the homogeneity assumption. However, contrary to [15] and [6], in this work we are not restricted to decreasing with time users’ interests, but we apply diverse function forms of the latter (e.g., periodic).

Apart from analyzing the spreading, controlling the information diffusion over various types of networks via explicit, e.g., [7, 10, 20–22] or implicit control, e.g., [16], is highly important. A thorough overview of the current framework of controlling epidemics can be found in [7], including heuristic feedback methods and optimal control policies for both population dynamics and deterministic network models along with spectral control policies for the latter. The authors usually adopt optimal control frameworks for obtaining features allowing the control of the corresponding diffusion properties, which are modeled via differential equations (deterministic models). The work in [20], studies the possible attack strategies of malware over wireless networks and the extent of damage they can sustain. The control parameters consist of the transmission range and media scanning rate of the worm targeting at accelerating its spread. Malware information dissemination is also studied in [10], where the control signal distribution time is determined, aiming to minimize the number of infected nodes and the cost of control. Similar approaches for malware quarantining and filtering (e.g., configurable firewall) are developed and analyzed in [21–23]. These problem approaches, although different in various scopes compared to the target of this paper, they resemble and serve as driving forces to our proposed model and analysis.

Considering implicit control, in [16], malware information propagation is studied and analyzed over a homogeneous mixing network, where control takes the form of updates to nodes from an external source to which nodes reply via a best response (game theoretic) scheme. Also, in [16], there is an implicit introduction (i.e., via the time-varying state of the system) of time-varying behavior on the parameters of the information diffusion epidemic model. However, this takes place in a more restricted sense and with a different scope (i.e., malware propagation) compared to our work.

Our work dealing with non-malicious information, identifies a major driving force for the successfulness of diffusion, namely user interest and its temporal properties, opening up new directions for the optimal control of useful information diffusion taking into account these aspects as well. Moreover, although our approach adopts a similar problem formulation and analytical approach as in [20], contrary to [7, 10, 20–22], the state constraint, i.e., the epidemic-based differential equation of the evolution of the number of infected nodes, has time-varying parameters due to the temporal dependence of users’ interests considered in this paper.

## System model, notation and assumptions

**Fig. 1:**
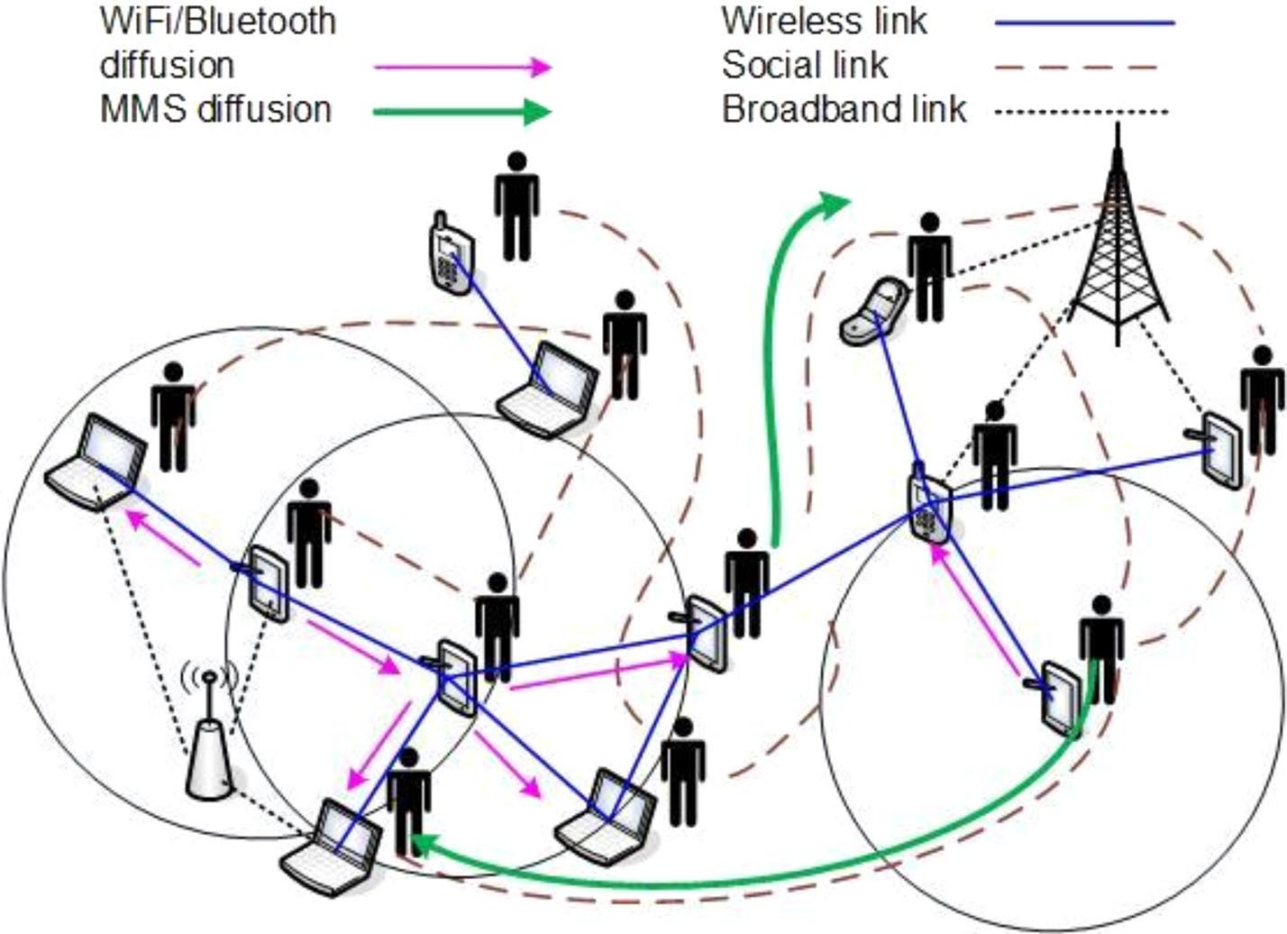
The considered information diffusion mechanisms over generalized networks. WiFi/Bluetooth diffusion (*purple arrows*) includes all neighbors within the transmission range of the user (physical layer), while MMS diffusion (*green arrows*) may take place with only specific neighbors of a user in social layer depending on their interest values in the propagated information

We focus on information diffusion and its optimal control in generalized networks, where the substrate is a wireless multihop network, i.e., user devices. Two different spreading pathways develop in such networks, namely information diffusion via either Multimedia Messaging Service (MMS) in the social layer or via WiFi/bluetooth (P) in the physical substrate [1] (Fig. 1). The former acts as a “long-range” information spread, since nodes communicating directly at the social layer may be actually separated by many hops in the physical layer. MMS transfers act as diffusion shortcuts. On the contrary, P-type information transfers act as local information ripples over short-range areas around information processors.

We consider a wireless multihop network of *N* nodes uniformly and independently distributed on a square region of side *L*. Each node has a transmission radius *R*. For simplicity, mobility is ignored, since compared to an MMS type of information spreading, the corresponding long-range information spreading achieved by mobility, which is essentially of P type, will lead to a similar, but smaller effect, as also argued in [1].

We assume *M* classes of information denoted by $m=1...M$, as in [24]. Each class consists of messages on a specific topic, e.g., summer vacations advertisements. Information diffusion is studied separately for each class; however, interactions among separate classes are taken into consideration in the information diffusion model’s probabilistic setting. Each node *i* is characterized by its interest in class *m* at time *t*, denoted as $R_i^m(t)$, where $\sum _{\forall m} R_i^m(t)\le 1, ~\forall i$ (e.g., normalization over all classes). The information diffusion process proposed in this paper is based on the Susceptible–Infected–Susceptible (SIS) epidemic model [9]. We consider the following mapping. A node *i* is considered Infected (i.e., informed) for a specific class *m* of information if it possesses at least one message belonging in this class, otherwise *i* is considered Susceptible (i.e., not informed) for class *m*. This means that an informed/not-informed node is mapped to an infected/susceptible state correspondingly, in epidemiology terms. More precisely, the transition from the susceptible state to the infected state for a particular class takes place when a node receives information about this class, while an infected node transits back to the susceptible state when it deletes all messages for this class.

In the rest of the paper, we will employ the notation provided in Table 1. If the network is directed, the out-degree is considered. $f_1(x), f_2(x), f_3(x)$ are general functions that will be used in the information diffusion model. Finally, the system model will be further enhanced in "Optimal control framework for information diffusion", where the optimal control framework over the information dissemination modeling framework in generalized networks is introduced.

**Table 1:** Notation and explanation of symbols.

Symbol	Interpretation
$I^m(t)$	Number of Infected nodes for class *m*
$S^m(t)$	Number of Susceptible nodes concerning class *m*
$\mathcal {I}^m(t)$	Set of Infected nodes concerning class *m*
$\mathcal {N}_S(i)$	Set of node *i*’s friends in the social layer
$\mathcal {N}_{P}(i)$	Set of connections of *i* in the wireless network (physical layer)
$0\le p_1,p_2,q\le 1$	Probabilities defined in the proposed information diffusion model
$N_S^{avg}$	The average degree of all nodes in the social layer
$f_1(x)$	$f_1(x):[0,1]\rightarrow [0,1]$ monotonically increasing on *x*
$f_2(x)$	$f_2(x):[0,1]\rightarrow [0,1]$ monotonically decreasing on *x*
$f_{3}(x)$	$f_{3}(x):[0,1]\rightarrow [0,1]$ monotonically increasing on *x*

## Information diffusion modeling and analysis without control

In this section, we describe the proposed information diffusion process that considers users’ social features/interests. Its analysis via epidemic modeling leads to the incorporation of the impact of nodes’ interests on the information diffusion dynamics. With respect to information diffusion, a node *i* is expected to perform/experience one of the following actions at each time *t*.

1. For the classes for which *i* is infected/informed:
  - *i* diffuses information about class *m* with probability $f_1(R_i^m(t))$ (also denoted as $f_1(t)$ for simplicity),
  - *i* deletes all messages about class *m* with probability $q f_2(R_i^m(t))$, where the parameter *q* is introduced to control the deletion process and $f_2(R_i^m(t))$ will be also denoted as $f_2(t)$ for simplicity.
  We consider that the duration of each time slot permits only the completion of one action, thus only one class *m* will be selected and either (a) or (b) will happen. This means that
  $$\begin{aligned} \sum _{m:~i\in \mathcal {I}^m(t)} (f_1(R_i^m(t))+q f_2(R_i^m(t)))\le 1. \end{aligned}$$
2. Node *i* performs another action—which is not of interest for the information diffusion—with probability equal to $1-\sum _{m:~i\in \mathcal {I}^m(t)} (f_1(R_i^m(t))+q f_2(R_i^m(t)))$.

If choosing one class *m* for action (a) (with probability $f_1(R_i^m(t))$), node *i* performs one of the following:

- with probability $p_1$, node *i* employs an MMS type of transmission, including as receivers each $j\in \mathcal {N}_S(i)$ selected with probability $f_{3}(R_j^m(t))$ (also denoted as $f_3(t)$ for simplicity), where $f_{3}(R_j^m(t))$ for all $j\in \mathcal {N}_S(i)$ does not form a probability distribution,
- with probability $p_2$, node *i* broadcasts to all its $\mathcal {N}_{P}(i)$ neighbors (*P*-type action).

Note that $$p_1+p_2\le 1.$$

The above diffusion process requires that there is always an infected node for every class to maintain the spreading. However, all infected nodes for a particular class may delete their information for this class, thus disrupting its diffusion. Exogenous impact such as the optimal control which will be introduced in "Optimal control framework for information diffusion", may be leveraged to alleviate in a certain degree such phenomena of extinction of a whole information class. In the case of P-type contacts, we do not consider the interests of users receiving a P-induced message, as the latter is broadcasted indiscriminately to all of them.

In the following, we model the dynamics of the evolution of the number of infected nodes for each class *m*, $I^m(t)$, via differential equations that approximate the system evolution. Specifically, the approximate dynamics of $I^m(t)$ are captured by the following ordinary differential equation (ODE),

1 $$\begin{aligned} \frac{\mathrm{d}I^m(t)}{\mathrm{d}t}&= p_1 f_{1}^{\mathrm{avg}}(t) f_{3}^{\mathrm{avg}}(t) \frac{S^m(t)}{N} I^m(t)N_s^{\mathrm{avg}}\nonumber \\&\quad +p_2 N\frac{\pi R^2}{L^2}\frac{S^m(t)}{N} I^m(t)f_{1}^{\mathrm{avg}}(t)\nonumber \\&\quad -q I^m(t)f_{2}^{\mathrm{avg}}(t), \end{aligned}$$

where $f_{3}^{\mathrm{avg}}(t)$ is the average or the expected value of all $f_{3}$ functions over the network at time *t* for the corresponding class *m*. Functions $f_{1}^{\mathrm{avg}}(t), ~f_{2}^{\mathrm{avg}}(t)$ are similarly defined. The initial conditions are $I^m(0)=I_0^m, \forall m$, i.e., $I_0^m$ nodes are initially infected for each information class *m* via the social layer.

The ODE (1) has a unique solution when $f_{1}^{\mathrm{avg}}(t),~f_{2}^{\mathrm{avg}}(t), ~f_{3}^{\mathrm{avg}}(t)$ are continuous functions with respect to time (Cauchy–Lipschitz Theorem [25]). The right-hand side is obviously Lipschitz continuous with respect to $I^m$. This fact has an impact on the design of possible forms for the interests’ functions $R_i^m(t),~\forall m,i$, which should be continuous in time. It also has impact on the design of possible formats for the functions $f_{1}^{\mathrm{avg}}(t),~f_{2}^{\mathrm{avg}}(t), ~f_{3}^{\mathrm{avg}}(t)$.

A suitable selection for the functions $f_1,f_2,f_{3}$ in the working example scenarios that follow is:

2 $$\begin{aligned} f_1(R_i^m(t))&=\frac{R_i^m(t)}{2 M},\nonumber \\ f_2(R_i^m(t))&=\frac{1-R_i^m(t)}{2 M}, \nonumber \\ f_{3}(R_j^m(t))&=R_j^m(t). \end{aligned}$$

This configuration is not restrictive in the sense that others may be designed for other scenarios/applications. It is important to note that Eq. (1) is approximate since averages or expected values of the functions $f_1,f_2,f_{3}$ are used. However, such an approximate form can be used to demonstrate the important characteristics of information diffusion dynamics in specific interesting cases that will be examined via appropriately designed scenarios in the sequel.

### Scenario 1: periodic users’ interests

In this scenario, two classes of information are considered. The time continuous interests’ functions take sinusoidal forms to express users’ time periodicity of their interest with respect to the propagated information. Specifically, $R_i^1(t)=1-A_i\sin ^2(a(t+b_i))+B_i, ~\forall i,$ for class $m=1$, where $a>0$ determines the period of users’ interests and $A_i,~b_i,~B_i$ are appropriately defined constants. Then, $R_i^2(t)=A_i\sin ^2(a(t+b_i))-B_i, ~\forall i$, so that $R_i^1(t)+R_i^2(t)=1,~\forall t,i$. Note that, we consider the same frequency for all sinusoidal interests assuming the propagation of information that intrigues the attention of all users over specific time periods such as vacations, summer sports, Halloween, etc.

Based on the configuration for the functions $f_1,f_2,f_{3}$ defined above (Eq. 2), their average values for class 1 become

$$\begin{aligned} f_{1}^{\mathrm{avg}}(t) = &\, \frac{1-A\sin ^2(a(t+b))+B}{2 M},\\ f_{2}^{\mathrm{avg}}(t) = &\, \frac{A\sin ^2(a(t+b))-B}{2 M},\\ f_{3}^{\mathrm{avg}}(t) = &\, 1-A\sin ^2(a(t+b))+B, \end{aligned}$$

where $b_i=b,~\forall ~i,$ and the constants *A*,  *B* are computed by averaging the interests over all users at time *t*. The average values of functions $f_1,f_2,f_{3}$ for class 2 are defined similarly.

We can also assume that $f_{1}^{\mathrm{avg}}(t), ~f_{2}^{\mathrm{avg}}(t),~f_{3}^{\mathrm{avg}}(t)$ represent the expected values of the corresponding functions of users’ interests at time *t*. Thus, users’ interests will vary randomly according to a distribution with mean value $1-A\sin ^2(a(t+b))+B$ for class 1, letting the complementary interest (i.e., with mean value $A\sin ^2(a(t+b))-B)$ to be assigned to class 2.

In this case, the ODE (1) for class 1, becomes

3 $$\begin{aligned} \frac{\mathrm{d}I^1(t)}{\mathrm{d}t}= & \, \frac{p_1 N_s^{\mathrm{avg}}}{N} (N-I^1(t))I^1(t)\frac{(1-A\sin ^2(a(t+b))+B)^2}{2M}\nonumber \\&\quad + p_2\frac{\pi R^2}{L^2} (N-I^1(t)) I^1(t)\frac{(1-A\sin ^2(a(t+b))+B)}{2M}\nonumber \\&\quad -q I^1(t)\frac{(A\sin ^2(a(t+b))-B)}{2M}, \end{aligned}$$

and similarly, the ODE (1) for class 2, can be written as

4 $$\begin{aligned} \frac{\mathrm{d}I^2(t)}{\mathrm{d}t}= & \, \frac{p_1 N_s^{\mathrm{avg}}}{N} (N-I^2(t))I^2(t)\frac{(A\sin ^2(a(t+b))-B)^2}{2M}\nonumber \\&\quad +p_2\frac{\pi R^2}{L^2} (N-I^2(t)) I^2(t)\frac{(A\sin ^2(a(t+b))-B)}{2M}\nonumber \\&\quad -q I^2(t)\frac{(1-A\sin ^2(a(t+b))+B)}{2M}. \end{aligned}$$

The solution of both Eqs. (3), (4) takes a complex form which does not provide any intuition regarding the dynamics of change of the number of infected nodes for each class, $I^1(t),~I^2(t)$. For this reason, we apply a finite difference approach to approximate them as follows. Let $M_1(t)$ be the right-hand side of Eq. (3) and $M_2(t)$ be the right-hand side of Eq. (4). Then, the finite difference scheme with sufficiently small time step $\Delta t>0$ and $t\ge 0$ yields:

5 $$\begin{aligned} I^1(t+\Delta t)=I^1(t)+M_1(t)\cdot \Delta t, \end{aligned}$$

6 $$\begin{aligned} I^2(t+\Delta t)=I^2(t)+M_2(t)\cdot \Delta t. \end{aligned}$$

It can be observed that when $1-A\sin ^2(a(t+b))+B\cong 0$, $M_1(t)\cong -\frac{q I^1(t)}{2M}$, thus $I^1(t+\Delta t)< I^1(t)$ for that time periods, while complementarily $S^1(t+\Delta t)> S^1(t)$. The converse holds for the time periods where $1-A\sin ^2(a(t+b))+B\cong 1$. Therefore, the periodicity of user interests is reflected in the information diffusion dynamics, where it is possible that the number of infected nodes does not converge to a specific value but rather fluctuates according to a time period determined by user interest periodicity.

### Scenario 2: comparison of information diffusion dynamics among groups with different characteristics

In this scenario, we apply constant interests to study how information of a specific subject spreads in groups characterized by different features such as in the second example described in the introductory section ("Background"). This special case is similar to the SIS models developed in literature [8, 9] in the sense that the parameters applied in the ODEs describing the dynamics of information diffusion are constant, contrary to the time varying parameters ($f_{1}^{\mathrm{avg}}(t),~ f_{2}^{\mathrm{avg}}(t),~ f_{3}^{\mathrm{avg}}(t)$) considered in this paper. Therefore, the already existing schemes [26] constitute special cases of our proposed diffusion model.

In this framework, we consider two groups and one information class (e.g., class 1). For both groups $R_i^1(t)=a, ~\forall i$, $0<a<1$, where for the first group *a* is close to 1 while in the second group *a* gets closer to 0. In this particular case of constant interests, the solution of Eq. (1) attains a less complex form than in Scenario 1. However, we will use again the finite difference approximation of Eqs. (5), (6), where the definitions of $M_1(t),~M_2(t)$ are based on constant interests adapted for the two groups correspondingly, to get more intuition about the derived convergence in the number of infected nodes. Specifically, as it will be verified via simulation and numerical results in "Simulation and numerical results without applying control", a higher constant interest by users implies convergence of the number of infected nodes to a higher value.

### Scenario 3: increasing vs. decreasing users’ interest

In this scenario, there exist two classes of information, while the population has increasing interest for the one class and decreasing for the other. The appropriate interest functions for this case are formulated, $\forall i$, as follows:

7 $$\begin{aligned} R_i^1(t)=B\frac{At}{At+C},~ R_i^2(t)=B\frac{C}{At+C}, \end{aligned}$$

where *A*,   *B*,   *C* are constants.

Again, we will use the finite difference approximation of Eqs. (5), (6), where the definitions of $M_1(t),~M_2(t)$ for each class correspondingly are based on Eq. (7).

## Optimal control framework for information diffusion

In this section, we introduce an optimal control framework for the previously presented information diffusion model for a specific class *m*. The objective in this optimal control problem is to maximize the number of infected (informed) nodes for a topic/class *m* by applying an exogenous aid/force, i.e., the control, while taking into account associated control costs, e.g., advertising cost. The motivation behind this is twofold. First it might be necessary to apply a control action to boost users’ interest to increase information spreading. Secondly, more resources might be required (by increasing a control signal) when users are more interested in a topic to conserve resources by not wasting them when users are not interested in the propagated information. Thus, this approach will allow affecting the information diffusion over the susceptible (non-informed) users, via properly controlling user interests.

Assuming the control signal is given by a function $u(.)=\{u(t) |t \in [0,T]\}$, we aim at maximizing the objective function:

8 $$\begin{aligned} J(u(.))=\int _0^T{\left( k_1I^m(t)+k_2u(t)\right) \mathrm{d}t}+k_II^m(T), \end{aligned}$$

where $k_1,k_I\ge 0$, $k_2\le 0$ are parameters expressing the trade-off between control cost and diffusion efficiency. Parameters $k_1,k_2$ refer to the operation during a specific time interval within [0, *T*] and $k_I$ refers to the final state of the system. We aim at finding an optimal control $u^*(.)$ such that:

9 $$\begin{aligned} J(u^*(.))=\max _{u(.)}J(u(.)). \end{aligned}$$

The control problem will be solved subject to the approximate dynamics of the evolution of the number of informed nodes, which is similar to Eq. (1):

10 $$\begin{aligned} \frac{\mathrm{d}I^m(t)}{\mathrm{d}t}= & \, \frac{N_s^{\mathrm{avg}}p_1}{N} f_1^{\mathrm{avg}}(t) f_3^{\mathrm{avg}}(t) (N-I^m(t)) I^m(t) g_1(u(t))\nonumber \\ & +p_2 \frac{\pi R^2}{L^2} f_{1}^{\mathrm{avg}}(t) I^m(t) (N-I^m(t))g_1(u(t)) \nonumber \\&\quad -q I^m(t)f_{2}^{\mathrm{avg}}(t) g_2(u(t)), \end{aligned}$$

with $I^m(0)=I_0^m$ ($S^m(0)=N-I_0^m$). Also the following state conditions should hold:

11 $$\begin{aligned} N=I^m(t)+S^m(t),~ 0\le I^m(t)\le N,~ t \in [0,T], \end{aligned}$$

for every *t*. Note that Eq. (10) differs from Eq. (1) due to the introduction of the control *u*(*t*) in the summands of its right-hand side, where the probabilities $f_1^{\mathrm{avg}}(t),~ f_2^{\mathrm{avg}}(t)$ have been replaced with $f_1^{\mathrm{avg}}(t) g_1(u(t)),~f_2^{\mathrm{avg}}(t) g_2(u(t))$. The control *u*(.) depends on the controller’s budget for topic *m*, while the control region is defined as $\Omega =\{u(.):[0,T]\rightarrow \mathfrak {R}| u_{\min }\le u(t)\le u_{\max },~\forall ~t \}$ and also each *u*(.) is a piece-wise continuous function such that its left and right limits exist. Functions $g_1, g_2$ are non-negative, differentiable and either convex or concave with respect to *u*. While $g_1$ is increasing with *u*, $g_2$ is decreasing, and $g_1,g_2:[u_{\min },u_{\max }]\rightarrow [0,1]$. The control might take the form of incentives for increasing user interest, or it may exploit the increased user interest to reinforce information spreading, while such behavior will be explored in the following.

The next proposition allows us to ignore the state constraints expressed in Eq. (11) in the rest of the analysis.

### **Proposition 1**

*For any* $u(.)\in \Omega$ *, the state function* $I^m(.):[0,T]\rightarrow \mathfrak {R}$ *that satisfies* $I^m(0)=I_0^m$ *, also satisfies Eq.* (11).

### *Proof*

Let $t_0$ be the first time instant in [0, *T*] where $I^m(t_0)=0$ or $I^m(t_0)=N$.

- If $I^m(t_0)=0$ then $S^m(t_0)=N$ and $\frac{\mathrm{d} I^m(t)}{\mathrm{d} t}|_{t=t_0^{+}}=0$, meaning that $I^m(t)=0$, for every $t>t_0$, $t\le T$.
- If $I^m(t_0)=N$ then $S^m(t_0)=0$. Thus, $\frac{\mathrm{d} I^m(t)}{\mathrm{d} t}|_{t=t_0^+}=-q N \, f_2^{\mathrm{avg}}(t_0)g_2(u(t_0))<0$, since $f_2^{\mathrm{avg}}(t_0),g_2(u(t_0))>0$, meaning that $I^m(t_0^+)\le N$. Similarly for all other $t^{\prime } \in (t_0,T]$ where $I^m(t^{\prime })=N$.

$$\square$$

The following proposition proves that the number of infected nodes for class *m*, $I^m(t)$ is strictly positive for every $t \in [0,T]$.

### **Proposition 2**

*We have that* $I^m(t)\ge I_0^m e^{-q f_{2_{\max }}^{avg}g_2(u_{\min })t}\ge 0$, $\forall t\in [0,T]$.

### *Proof*

It holds that $\frac{\mathrm{d} I^m(t)}{\mathrm{d} t}\ge -q I^m(t)f_2^{\mathrm{avg}}(t)g_2(u(t))$, which means that $\frac{I^m(t)^{\prime }}{I^m(t)}\ge -q f_{2{\max }}^{\mathrm{avg}}g_2(u_{\min })$, where $f_{2\max }^{\mathrm{avg}}=\max _{\forall t} f_2^{\mathrm{avg}}(t)$ and since $g_2$ is decreasing with *u*. Thus, $\ln I^m(t)\ge -q f_{2_{\max }}^{\mathrm{avg}} g_2(u_{\min })t+\ln I^m(0)$, yielding $I^m(t)\ge I_0^m e^{-q f_{2_{\max }}^{\mathrm{avg}} g_2(u_{\min }) t}$, for every $t\in [0,T]$. $\square$

### **Definition 1**

The pair $(I^m(.),u(.))$ is an admissible pair if the following hold: (i) $u(.)\in \Omega$, (ii) the state $(I^m(.))$ constraint of Eq. (10) holds. Then *u*(.) is called an admissible control [20].

### **Definition 2**

An admissible control *u*(.) is an optimal control, if $J(u(.))\ge J(\underline{u}(.))$ for all admissible controls $\underline{u}(.)$ [20].

Based on these two definitions, we will apply Pontryagin’s Maximum Principle [20, 27–29] to determine the optimal control’s functional form and study its properties. Let us denote as $\lambda (t)$ the adjoint/costate variable of the Pontryagin’s Maximum Principle [27] at time $t \in [0,T]$. First, we define the Hamiltonian function, $\mathcal {H}$, at time *t* as:

12 $$\begin{aligned} \mathcal {H}(I^m(t),u(t),\lambda (t))&=k_1 I^m(t) + k_2 u(t) \nonumber \\&\quad + \lambda (t)\left[ \frac{N_s^{\mathrm{avg}}p_1}{N} f_1^{\mathrm{avg}}(t) f_3^{\mathrm{avg}}(t) (N-I^m(t)) I^m(t) g_1(u(t))\right. \nonumber \\&\quad +p_2 \frac{\pi R^2}{L^2} f_{1}^{\mathrm{avg}}(t) I^m(t) (N-I^m(t))g_1(u(t)) \nonumber \\&\quad \left. - \, q I^m(t)f_{2}^{\mathrm{avg}}(t) g_2(u(t))\right] . \end{aligned}$$

Assuming that $u^*(.)$ is the optimal control value and $I^{m^*}(.)$ is the corresponding state trajectory, i.e., the one solving Eq. (10) for $u^*(.)$, according to Pontryagin’s Maximum Principle [27], there exists a function $\lambda ^*(.):[0,T]\rightarrow \mathfrak {R}$ such that

13 $$\begin{aligned} \frac{\mathrm{d} \lambda ^*(t)}{\mathrm{d}t}&=-\frac{\partial \mathcal {H}}{\partial I^m}=-k_1- \lambda ^*(t)\left[ \frac{N_s^{\mathrm{avg}}p_1}{N} f_1^{\mathrm{avg}}(t) f_3^{\mathrm{avg}}(t) (N-2I^{m*}(t)) g_1(u^*(t))\right. \nonumber \\&\quad \left. + \,\, p_2 \frac{\pi R^2}{L^2} f_{1}^{\mathrm{avg}}(t) (N-2I^{m^*}(t))g_1(u^*(t)) \right. \nonumber \\&\quad \left. \vphantom{\frac{N_s^{\mathrm{avg}}p_1}{N} f_1^{\mathrm{avg}}(t) f_3^{\mathrm{avg}}(t) (N-2I^{m*}(t)) g_1(u^*(t))} - \,q f_{2}^{\mathrm{avg}}(t) g_2(u^*(t))\right] ,\end{aligned}$$

14 $$\begin{aligned} \lambda ^*(T)=k_I (\text {transversality condition}), \end{aligned}$$

where $\lambda ^*(.)$ is the optimal costate (adjoint) function and also the optimal control, $u^*(.)$, is computed as:

15 $$\begin{aligned} u^*(t)=u(t)\in \arg \max _{\underline{u}\in \Omega }\mathcal {H}(I^{m^*}(t),\underline{u},\lambda ^*(t)),~ 0\le t\le T. \end{aligned}$$

For simplicity, from now on we omit the symbol $^*$ from the optimal values. We can prove the following proposition which will be useful in studying the properties of the optimal control function.

### **Proposition 3**

*We have that* $\lambda (t)>0$ *for* $t\in [0,T)$.

### *Proof*

We follow a similar proof to the one of Lemma 2 in [20]. First we show that $\lambda (t)$ is strictly positive over an interval of non-zero length towards the end of [0, *T*). It holds that $\lambda (T) = k_I \ge 0$. If $k_I > 0$, this statement holds due to continuity. If $k_I = 0$, then from (13) and for $t=T$ we have: $\frac{d\lambda (t)}{dt}|_{t=T} = - k_1 <0$, i.e., descending from positive values before reaching the value $k_I=0$, and this statement also holds.

As $t^{\prime }< T$, consider the latest time in [0, *T*) that $\lambda (t^{\prime })=0$, i.e., $\lambda (t)>0$ for $t^{\prime }<t<T$. Then, $\frac{d\lambda (t)}{dt}|_{t=t^{{\prime }+}} = -k_1 < 0$ which is impossible since for $t>t^{\prime }$, $\lambda$ is positive and thus it should increase from the zero value. The latter statement concludes the proof of Proposition 3. $\square$

Let us now omit the time dependence over the employed notation for brevity reasons. We can define the functional:

16 $$\begin{aligned} \phi (u) &= k_2 u + \lambda \left[ \frac{N_s^{\mathrm{avg}}p_1}{N} f_1^{\mathrm{avg}} f_3^{\mathrm{avg}} (N-I^m) I^m g_1(u)\right. \nonumber \\ & \left. + \,\, p_2 \frac{\pi R^2}{L^2} f_{1}^{\mathrm{avg}} I^m (N-I^m)g_1(u) \right. \nonumber \\ & \left. \vphantom {\frac{N_s^{\mathrm{avg}}p_1}{N} f_1^{\mathrm{avg}} f_3^{\mathrm{avg}} (N-I^m) I^m g_1(u)} - \, q I^mf_{2}^{\mathrm{avg}} g_2(u)\right], \end{aligned}$$

and search for an optimal *u*, i.e., such that $\phi (u)\ge \phi (\underline{u})$ for all admissible $\underline{u}\in \Omega$. The functional $\phi (u)$ is derived by the Hamiltonian (Eq. 12) for a particular time, considering only the terms that depend on *u*.

Given the possible forms of the functions $g_1,g_2$ with respect to the control *u* (convex or concave), the following cases can be identified:

1. $g_1$ convex and $g_2$ concave. Then $\phi$ is convex with respect to *u*. Due to convex maximization, the optimal control will be necessarily at the extrema of the range of the control, determined by comparison as:
  17 $$\begin{aligned} u^* = \left\{ \begin{array}{rl} u_{\min } &{} \text{ if } \phi (u_{\min })>\phi (u_{\max }), \\ u_{\max } &{} \text{ if } \phi (u_{\min })<\phi (u_{\max }). \end{array} \right. \end{aligned}$$
2. $g_1$ concave and $g_2$ convex. Then $\phi$ is concave with respect to *u*. In this case, a concave maximization takes place, where the maxima of $\phi (u)$ occur at the points where the partial derivative with respect to *u* is zero, or at the extrema of the range of the control, determined by comparison. The equation $\frac{\partial \phi }{\partial u}=0$ becomes:
  18 $$\begin{aligned} -\frac{k_2}{\lambda } &= \frac{\partial g_1(u)}{\partial u} \left[ \frac{N_s^{\mathrm{avg}}p_1}{N} f_1^{\mathrm{avg}}f_3^{\mathrm{avg}} (N-I^m) I^m \right. \nonumber \\ & \left. + \,\, p_2 \frac{\pi R^2}{L^2} f_{1}^{\mathrm{avg}} I^m (N-I^m)\right] \nonumber \\ & -\frac{\partial g_2(u)}{\partial u} q I^m f_{2}^{\mathrm{avg}}, \end{aligned}$$
  where $\lambda >0$ from Proposition 3. If $u^{\prime }$ is the solution of the above, then the optimal control becomes:
  19 $$\begin{aligned} u^*=\max \left\{ u_{\min },\min \{u^{\prime },u_{\max }\}\right\} . \end{aligned}$$
  In this case based on the explicit forms of functions $g_1,~g_2$, we can study possible relations/properties of the optimal control with respect to users’ interests, as it will be performed in the following sections.
3. $g_1$ concave and $g_2$ concave. We have
  20 $$\begin{aligned} \frac{\partial ^2 \phi (u)}{\partial u^2} &= \lambda \left[ \frac{N_s^{\mathrm{avg}}p_1}{N} f_1^{\mathrm{avg}}f_3^{\mathrm{avg}} (N-I^m) I^m \frac{\partial ^2 g_1(u)}{\partial u^2}\right. \nonumber \\ &\left. +p_2 \frac{\pi R^2}{L^2} f_{1}^{\mathrm{avg}}I^m (N-I^m) \frac{\partial ^2 g_1(u)}{\partial u^2}\right. \nonumber \\ &\left. \vphantom {\frac{\partial ^2 g_1(u)}{\partial u^2}} - q I^m f_{2}^{\mathrm{avg}}\frac{\partial ^2 g_2(u)}{\partial u^2}\right] , \end{aligned}$$
  where $A_1 = \frac{N_s^{\mathrm{avg}}p_1}{N} f_1^{\mathrm{avg}}f_3^{\mathrm{avg}} (N-I^m) I^m \frac{\partial ^2 g_1(u)}{\partial u^2}+p_2 \frac{\pi R^2}{L^2} f_{1}^{\mathrm{avg}}I^m (N-I^m) \frac{\partial ^2 g_1(u)}{\partial u^2}\le 0$ and $B_1 = q I^m f_{2}^{\mathrm{avg}}\frac{\partial ^2 g_2(u)}{\partial u^2}\le 0$. Thus, $\frac{\partial ^2 \phi (u)}{\partial u^2}\le 0$ if $|A_1|\ge |B_1|$, that leads to a concave maximization as in case 2 above, or $\frac{\partial ^2 \phi (u)}{\partial u^2}\ge 0$ if $|A_1|\le |B_1|$, that leads to a convex maximization as in case 1 above.
4. $g_1$ convex and $g_2$ convex. Then $\frac{\partial ^2 g_1(u)}{\partial u^2}\ge 0$, $\frac{\partial ^2 g_2(u)}{\partial u^2}\ge 0$. Thus, $\frac{\partial ^2 \phi (u)}{\partial u^2}\ge 0$ if $|A_1|\ge |B_1|$, that leads to a convex maximization as in case 1 above, or $\frac{\partial ^2 \phi (u)}{\partial u^2}\le 0$ if $|A_1|\le |B_1|$, that leads to a concave maximization as in case 2 above.

We should note that the controller applies one kind of control with aim to increase $I^m(t)$ trading-off cost, but this impacts in a different way each part of the information propagation equation (i.e., Eq. 10) via the functions $g_1(u),g_2(u)$.

At this point, we study the case 2 more extensively, by choosing a (non-restrictive) specific form for the control functions $g_1(u),~g_2(u)$, as follows:

21 $$\begin{aligned} g_1(u)= & \,1+\frac{\ln (1+u)}{\ln (1+u_{\max })},\nonumber \\ g_2(u)= & \, \frac{\ln (1+u_{\max })-\ln (1+u))}{\ln (1+u_{\max })}. \end{aligned}$$

This choice serves the purpose of boosting the number of infected (informed) nodes for the examined class by increasing probabilities of communicating/transferring knowledge via $g_1(u)>1$ in the first and second summands of the right-hand side of Eq. (10), and by decreasing probabilities of knowledge “deletion” via $g_2(u)<1$ in the last summand of the right-hand side of Eq. (10). Then, after computing $\frac{\partial g_1(u)}{\partial u},~\frac{\partial g_2(u)}{\partial u}$, replacing them in Eq. (18) and solving the latter, the optimal control takes the following formula:

22 $$\begin{aligned} u^*=\left\{ -1 -\frac{\lambda \Gamma }{k_2} \right\} _{u_{\min }}^{u_{\max }}, \end{aligned}$$

where $\left\{ ~\right\} _{u_{\min }}^{u_{\max }}$ expresses projection to $[u_{\min },u_{\max }]$ and

23 $$\begin{aligned} \Gamma &= \frac{1}{\ln (1+u_{\max })} \left[ \frac{N_s^{\mathrm{avg}}p_1}{N} f_1^{\mathrm{avg}}f_3^{\mathrm{avg}} (N-I^m) I^m +p_2 \frac{\pi R^2}{L^2} f_{1}^{\mathrm{avg}} I^m (N-I^m)\right. \nonumber \\ & \quad \left. \vphantom {\frac{\pi R^2}{L^2} f_{1}^{\mathrm{avg}} I^m (N-I^m)} + q I^m f_{2}^{\mathrm{avg}}\right] . \end{aligned}$$

At this point, we study some properties of $\Gamma$ (Eq. 23) that will assist in the interpretation of the observable behavior of the optimal control in "Simulation results, numerical results and discussion in controlled information diffusion". First, we study the dependence of $\Gamma$ on the values of interest for the examined class *m*, i.e., $R_{\mathrm{avg}}^m$. By considering Eq. (2) providing the types of $f_1^{\mathrm{avg}},~ f_2^{\mathrm{avg}},~ f_3^{\mathrm{avg}}$, $\Gamma$ is increasing with $R_{\mathrm{avg}}^m$, i.e., $\frac{\partial \Gamma }{\partial R_{\mathrm{avg}}^m}>0$ if

24 $$\begin{aligned} I^m<N-\frac{q}{\frac{N_s^{\mathrm{avg}} p_1 2 R_{\mathrm{avg}}^m}{N}+\frac{p_2 \pi R^2}{L^2}}=I_{TH}. \end{aligned}$$

Therefore, $\Gamma$ will become an increasing function of interest for class *m* only if the number of infected nodes for class *m* becomes less that the threshold $I_{TH}$. This behavior is also expected for the optimal control itself, $u^*$ (Eq. 22), since by Proposition 3 it holds $-\frac{\lambda }{k_2}>0$ (if ignoring the dependence of $\lambda$ on $R_{avg}^m$). This fact, which will be verified via numerical evaluations in "Simulation results, numerical results and discussion in controlled information diffusion", indicates the targeted trade-off between information spread and cost, while leveraging users’ interests for class *m*. If the number of infected nodes for class *m* is high enough, higher that $I_{TH}$, the optimal control saves resources when the corresponding user interest for class *m* increases. When the number of infected nodes falls below $I_{TH}$, the control increases with user interest, aiming to leverage from higher values of interest to drastically boost the number of infected nodes for class *m*. This behavior emerges also in the case of power control in a wireless channel, where high power values are optimal under good channel conditions, to exploit the maximum possible data transfer rates [30]. Since $I_{TH}$ (Eq. 24) is time varying, as $R_{\mathrm{avg}}^m$ evolves with time ("Information diffusion modeling and analysis without control"), the monotony of $\Gamma$ may also change with time. Generally, higher values of $R_{\mathrm{avg}}^m$ lead to a higher range of values of the number of infected nodes for which $\Gamma$ is increasing with interest for class *m*.

Obviously, $\Gamma$ is a concave function of $I^m$, attaining its maximum at $I^m_{\max }=\frac{F R_{\mathrm{avg}}^m+ G (\frac{1}{R_{\mathrm{avg}}^m}-1)+K}{D R_{\mathrm{avg}}^m+E}$, where $F=\frac{N_S^{\mathrm{avg}} p_1}{2M}$, $E=\frac{p_2 \pi R^2}{L^2 M}$, $D=\frac{N_S^{\mathrm{avg}} p_1}{MN}$, $G=\frac{q}{2M}$, $K=\frac{p_2 \pi R^2N}{L^2 2M}$. Note that $I^m_{\max }$ is decreasing with interest when $(FE +GD-DK)(R_{\mathrm{avg}}^{m})^2-2GD R_{\mathrm{avg}}^m-GE <0$, where *D*, *E*, *F*, *G*, *K*, are determined by the parameters of the system. In this case, for higher values of interest it is intuitively expected that the optimal control (Eq. 22) will achieve its maximum on a lower value of $I^m$, if ignoring any dependence of $\lambda$ on the examined parameters, i.e., $I^m, R_{\mathrm{avg}}^m$.

### Computing the optimal control value

Although Eqs. (17, 19) provide the form of the optimal control, computing the optimal control value is more complex, demanding the knowledge of the value of the adjoint variable, $\lambda$, for each *t*. In this section, we construct the Hamilton–Jacobi–Bellman (HJB) equation [27–29] and solve it via a numerical approach to obtain optimal control values within the control time interval ([0, *T*]).

#### **Definition 3**

We define the Value function $V(I^m,t)$, where $I^m(t)=I^m\in \mathfrak {R}, ~0\le t \le T$, as follows:

$$\begin{aligned} V(I^m,t)=\sup _{u(.) \in \Omega } J(u(.)), \quad V(I^m,T)=k_I I^m \, \text {(final condition)}. \end{aligned}$$

Actually, the Value function is obtained by varying the starting time of control within the control interval [0, *T*] and the initial value of infected nodes for class *m*. The HJB equation is formulated as the following partial differential equation:

25 $$\begin{aligned}& \frac{\mathrm{d} V(I^m,t)}{\mathrm{d} t}+ \max _{\underline{u} \in \Omega }\mathcal {H}\left( I^m,\underline{u},\frac{\partial V(I^m,t)}{\partial I^m}\right) =0,\nonumber \\ &V(I^m,T)=k_I I^m, \end{aligned}$$

where $\mathcal {H}$ is the Hamiltonian function defined in Eq. (12). Also, at each time $t \in [0,T]$, we have $\frac{\partial V(I^{m*}(t),t)}{\partial I^m}=\lambda ^*(t)$, where the symbol $^*$ again is used for denoting the optimal values obtained for $u^*(t)$. At this point, we will solve numerically the HJB equation [31] (Eq. 25) to compute the Value function for each $t\in [0,T)$. Then, the optimal control values will be obtained via Eqs. (17, 19) if replacing $\lambda$ with $\frac{\partial V}{\partial I^m}$ both computed at the examined time *t*. Applying a finite difference scheme and denoting as *u*(*t*) the optimal control value, Eq. (25) takes the following form:

26 $$\begin{aligned} &\frac{V(I^m,t)-V(I^m,t-\Delta t)}{\Delta t}+ k_1 I^m+ k_2 u(t)\nonumber \\ & \quad + \frac{V(I^m+\Delta I^m,t)-V(I^m-\Delta I^m,t)}{2\Delta I^m} \left[ \frac{N_s^{avg}p_1}{N} f_1^{avg}(t) f_3^{avg}(t) (N-I^m) I^m g_1(u(t))\right. \nonumber \\ & \quad + \left. p_2 \frac{\pi R^2}{L^2} f_{1}^{avg}(t) I^m(N-I^m)g_1(u(t)) -q I^m f_{2}^{avg}(t) g_2(u(t))\right] =0,\nonumber \\ &V(I^m,T)=k_I I^m, \end{aligned}$$

where $\Delta I^m, ~\Delta t$ are the steps of state and time and Eq. (26) is solved backwards since we know the Value function at the end, *T*, of the control time interval. Specifically, we compute the Value function for all $I^m \in \{1,2,...,N\}$ at time $0\le t-\Delta t < T$ from the corresponding ones at time *t*. When computing the optimal control values via Eqs. (19), (17) we replace $\lambda$ with $\frac{\partial V}{\partial I^m}\big |_t=\frac{V(I^m+\Delta I^m,t)-V(I^m-\Delta I^m,t)}{2\Delta I^m}.$ Furthermore, in the numerical solution of the HJB, we apply as a boundary condition for the partial derivative of the Value function with respect to state the following $\frac{\partial V}{\partial I^m}\big |_{t=T}=\frac{\partial V}{\partial I^m}\big |_{t=T-\Delta t}$.

## Simulation and numerical results without applying control

In this section, we present simulation and numerical results for each users’ interest scenario of "Information diffusion modeling and analysis without control". Specifically, the simulation results refer to the realization of the diffusion model described in "Information diffusion modeling and analysis without control" in MATLAB, while numerical results refer to the approximate solution (via finite difference scheme) of the ODEs in each scenario with the same parameters as in the corresponding simulation.

The simulation setting is as follows. We consider a generalized network, the wireless substrate of which consists of a wireless multihop network with $N=500$ nodes deployed over a square region with side $L=350m$ and with homogeneous transmission radius among nodes equal to $R=25m$. All simulation results are obtained as averages over several wireless topologies ($\#2$) and multiple repetitions ($\# 3$) for the diffusion at each topology. Furthermore, we examine two overlaying social network topologies over the same set of nodes as the wireless substrate, namely one scale-free and one small-world [32]. For the scale-free network topology, the social degree for each node is drawn from the power-law distribution with exponent 3, as observed for many social networks [33] (specifically the probability density function is $f(x)=\left( \frac{2}{x}\right) ^{3},~x\ge 2$), and the corresponding social layer’s neighbors of each node are chosen randomly. The small-world topology is constructed following the Watts & Strogatz paradigm [34]. For both topologies, $N_S^{\mathrm{avg}}\cong 4$. However, the degree distribution in the small-world topology is much more homogeneous than the corresponding one of the scale-free social topology. Also, the scale-free topology presents low average path length, which is a small-world feature [32]. Note that the value $\Delta t$ should be appropriately small, so that the solution of the ODE derived via the finite difference scheme (Eqs. 5, 6), approximates closely the precise solution of the ODE. We chose $\Delta t=0.4$. Finally, 10 nodes out of 500 are initially infected (e.g., via MMS) for each class in all simulation and numerical results that follow.

### Scenario 1: information diffusion dynamics in the case of periodic users’ interests

**Fig. 2:**
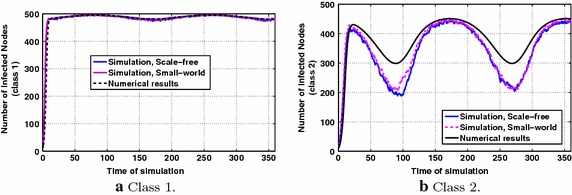
P & MMS types of information diffusion dynamics for periodic interests, with parameters $p_1=p_2=0.5$, $q=0.2$

In this scenario, we consider that the users’ interests vary uniformly and randomly with mean value $1-\frac{1}{4}\sin ^2(\frac{\pi }{180}(t+100))-\frac{1}{7}$ for the first class and $\frac{1}{4}\sin ^2(\frac{\pi }{180}(t+100))+\frac{1}{7}$ for the second class with the constraint that the interests of one node for the two classes are complementary (i.e., in the corresponding scenario of "Information diffusion modeling and analysis without control", $A=\frac{1}{4},~B=-\frac{1}{7},~a=\frac{\pi }{180},~b=100$). Therefore, the period of the interests’ functions is one year, a fact that can be reflected in realistic situations as the ones explained in "Background". The values of $p_1,~p_2,~q$ will be specified in each simulation case.

Fig. 2a compares the dynamics of the information diffusion for class 1 as derived by numerically solving Eq. (3) with the results obtained via simulations according to the proposed diffusion model in "Information diffusion modeling and analysis without control". The same is illustrated in Fig. 2b for class 2 (where the numerical results are obtained via numerically solving Eq. 4). The involved parameters take the values $p_1=p_2=0.5$, i.e., P and MMS types of transfer take place with the same probability in case of diffusion and $q=0.2$. The results are the same independently of the social topology, i.e., small-world or scale-free.

The periodic behavior of users’ interests is also reflected in the dynamics of information diffusion, as expected from the discussion in "Information diffusion modeling and analysis without control", and the number of infected nodes does not converge to a specific value as predicted by the conventional models in the literature [1, 13]. We observe that for class 1, the numerical results approximate well the ones obtained from simulations. In the case of class 2, i.e., in the case of lower interest for the information under propagation, it can be stated that the numerical results mostly overestimate the number of infected nodes. This observation can be explained by the fact that in the simulations, the number of infected nodes may become zero when all nodes delete their messages for a particular class, whereas according to Proposition 2, the theoretical number of infected nodes is always greater than zero. Thus, in the simulation it becomes likely that the information propagation for a class terminates (a fact that becomes more probable when interest values are lower), whereas in theory there is always enough quantity of infected nodes to spread information if the users’ interest for the latter increases.

**Fig. 3:**
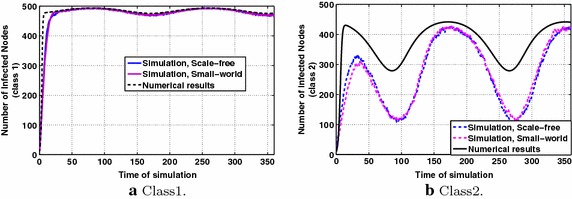
P type of information diffusion dynamics for periodic interests, with parameters $p_1=0,~p_2=1$, $q=0.6$

**Fig. 4:**
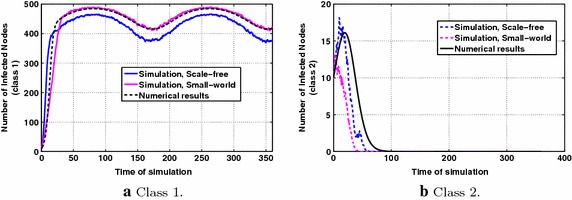
MMS type of information diffusion dynamics for periodic interests, with parameters $p_1=1,~p_2=0$, $q=0.6$

The results in Fig. 3a, b concern only the case of P type, while the results in Fig. 4a, b refer to the case of applying MMS type alone. By comparing these figures, it is observed that P type plays a significant role in maintaining the diffusion alive with respect to class 2 in which users’ interest for the diffused information attains lower values. Generally, P type further boosts information spreading for both classes. In Fig. 4a, for class 1, the diffusion dynamics over the small-world topology approximate much closer the numerical results than over the scale-free topology, whereas in the other cases, both topologies present similar behavior.

To conclude for this scenario, the theoretical model overestimates the volume of the information spreading, especially for class 2, which is characterized by lower values of interest. Also, the behavior in both cases of social topologies, i.e., small-world and scale-free, does not differentiate significantly.

### Scenario 2: information diffusion dynamics in the presence of groups with different characteristics

**Fig. 5:**
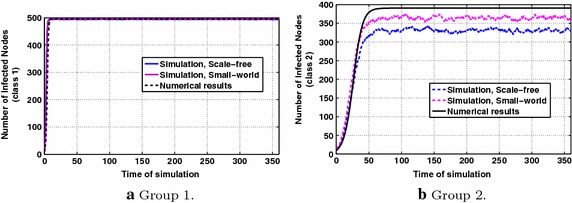
P & MMS types of information diffusion dynamics for constant interests, with parameters $p_1=0.6,~p_2=0.4$, $q=0.2$

In this evaluation scenario, based on the description of "Information diffusion modeling and analysis without control", we consider one information class and two groups with different interests in this information class. Group 1 has an interest value of 0.8 and Group 2 has an interest value of 0.2. Fig. 5a, b indicate that the interest plays significant role in the number of infected nodes to which the dynamics of information diffusion converge. In Group 1 the participants of which are highly interested in this information class, finally all nodes become infected. The parameters’ values are specified in the legends of the figures. It is also observed that for high interest the approximation of the theoretical model to the simulation results is satisfactory, while for lower interest, the theoretical model overestimates the simulation results. However, in the latter case (Fig. 5b), the diffusion dynamics over the small-world topology obtained via simulations lie much closer to the numerical results. In Fig. 5a, where interest values are higher, both social topologies present similar behavior concerning the information diffusion dynamics.

### Scenario 3: information diffusion dynamics in the case of increasing vs. decreasing users’ interests

**Fig. 6:**
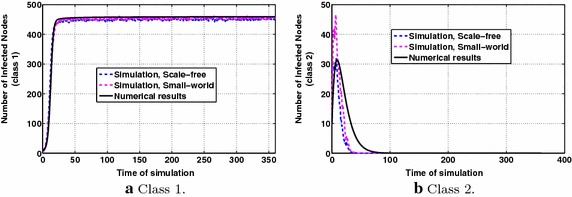
P & MMS types of information diffusion dynamics for decreasing vs. increasing with time interests, with parameters $p_1=0.5,~p_2=0.5$, $q=0.4$

The results regarding this evaluation scenario are shown in Fig. 6a, b, where $A=5,~C=10,~B=0.5$ in Eq. (7). Fig. 6a,  demonstrates the dynamics of information diffusion for the first class, where it is observed that the number of infected nodes initially presents a steep increase and then it increases with much lower rate. Regarding the information diffusion dynamics for class 2, from Fig. 6b it is observed that there is an initial increase which later deflates, due to the decreasing with time user interests, eventually yielding zero number of infected nodes for the simulation results and close to zero number of infected nodes for the numerical results (Proposition 2). Such dynamics cannot be captured by previous state-of-the art information diffusion models using constant diffusion parameters.

## Simulation results, numerical results and discussion in controlled information diffusion

In this section, we further study and evaluate the introduction of control—as described in "Optimal control framework for information diffusion"—in the three scenarios of "Simulation and numerical results without applying control". The values of the parameters that are used in "Simulation and numerical results without applying control" remain the same, except otherwise mentioned. Additionally, we consider $u_{\min }=0,~u_{\max }=30$, $\Delta I^m=1,~\forall ~m$, $\Delta t=10^{-4}$, $k_1=1,~k_2=-3,k_I=1$, $T=2$, and finally, the topology on the social layer is considered as scale free. Note that $\Delta t<< \Delta I^m$ so that the HJB solution converges [31]. The control is applied to only one class (or equivalently one group for constant interests) and specifically, we chose the class $m=2$ (or equivalently Group 2 for constant interests), to evaluate how information diffusion behaves under low values of interest when introducing control, and compare this behavior with the case when no control is applied (similarly to "Simulation and numerical results without applying control"). In the following subsections, in each scenario, we compare the numerical (derived via Eq. 10 using Eq. 21) and simulation results with and without control regarding the number of infected nodes for the second class/group, while we also study several properties and the behavior of the optimal control itself. We adapt the diffusion model of "Information diffusion modeling and analysis without control" to introduce control by replacing the probabilities $f_1^{\mathrm{avg}}(t),~ f_2^{\mathrm{avg}}(t)$ with $f_1^{\mathrm{avg}}(t) g_1(u(t)),~f_2^{\mathrm{avg}}(t) g_2(u(t))$, as implied by comparing Eq. (10) with Eq. (1). Every simulation runs for 200 time steps, i.e., the control time $T=2$ is divided into smaller time intervals each having a duration of 0.01.

### Scenario 1: controlled information diffusion dynamics in the case of periodic users’ interests

Similar to "Scenario 1: information diffusion dynamics in the case of periodic users’ interests", we consider that the users’ interests vary uniformly and randomly with mean value $1-\frac{1}{4}\sin ^2(\frac{\pi }{0.5}(t+0.2))-\frac{1}{7}$ for the first class and $\frac{1}{4}\sin ^2(\frac{\pi }{0.5}(t+0.2))+\frac{1}{7}$ for the second class with the constraint that the interests of one node for the two classes are complementary.

**Fig. 7:**
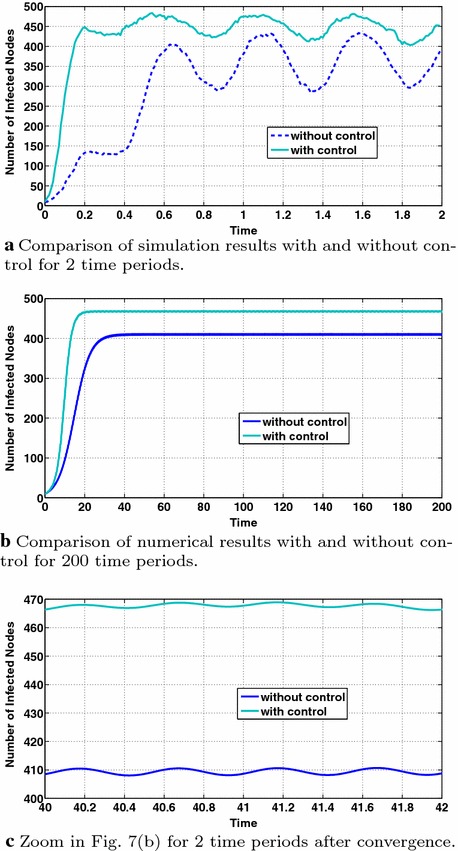
Periodic users’ interests: dynamics of information diffusion for class 2 with $p_1=p_2=0.5$, $q=0.3$

Fig. 7 presents the dynamics of the information diffusion for class 2 when applying periodic interests as defined above. Specifically, Fig. 7a depicts the dynamics of information diffusion for the second class derived from simulations with and without control. We observe that similar to the results of "Scenario 1: information diffusion dynamics in the case of periodic users’ interests" the periodicity of users’ interests is also reflected to the dynamics of information diffusion, while the introduction of control significantly improves the expected number of infected nodes (there is an increase of one hundred nodes) and reduces the amplitude of the sinusoidal curve, i.e., the variance of the number of infected nodes. Fig. 7b depicts the same comparison when the dynamics of information diffusion are derived via Eq. (10). Note that Eq. (10) is solved numerically in a similar way to Eq. (6), demanding more running time to converge due to the small value of $\Delta t$, which is the reason why the running time in Fig. 7b is more than two periods. Comparing Fig. 7a, b, we observe that the introduction of control leads to a much closer approximation of the simulation results from the numerical ones, compared to the absence of control that is also discussed in "Scenario 1: information diffusion dynamics in the case of periodic users’ interests". Finally, Fig. 7c zooms in two specific periods of Fig. 7b, after the convergence of the numerical solution of Eq. (10), to indicate in a clearer way the periodicity in the information diffusion dynamics.

**Fig. 8:**
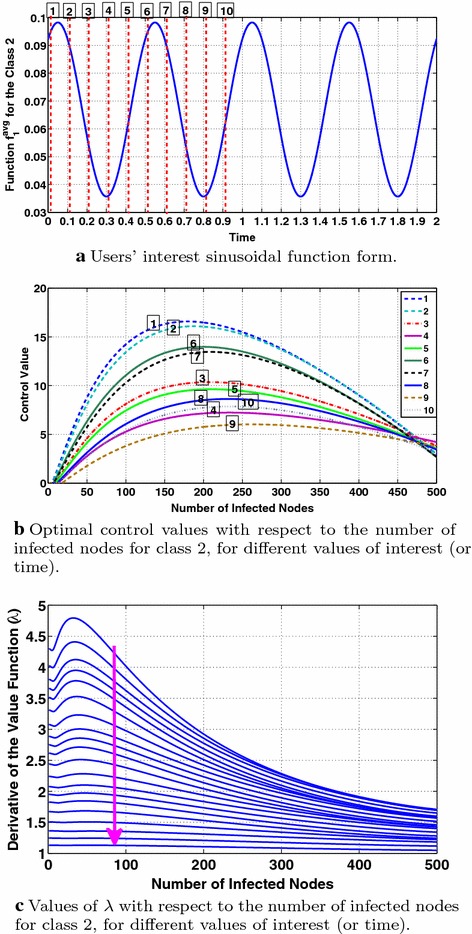
Study of the optimal control’s behavior for class 2. The *arrow* denotes time evolution

Figure 8 studies the behavior of the optimal control with respect to the number of infected nodes and the users’ evolving interest for the class $m=2$. From Fig. 8b, we observe that the optimal control is a concave function of the number of infected nodes (for all times), achieving its maximum value at a different number of infected nodes, $I_{\max }^m$, at each time or value of interest. This behavior is intuitively expected from the analysis of "Optimal control framework for information diffusion" with respect to $\Gamma$ (Eq. (23)), although it ignores the dependence of the adjoint variable $\lambda$ on the number of infected nodes. Furthermore, comparing Fig. 8a, b, we observe that $I_{\max }^m$ decreases with interest which can be intuitively explained by the fact that for our considered parameters the corresponding condition stated at the end of "Optimal control framework for information diffusion" is satisfied. More specifically, higher values of interest, e.g., points 1,  2,  6,  7 of the users’ interest sinusoidal function in Fig. 8a lead to lower values of $I_{\max }^m$ as it is observed in the corresponding optimal control curves 1,  2,  6,  7 in Fig. 8b. Moreover, comparing Fig. 8a, b and more specifically the interest values 1, 2, ..., 10 in Fig. 8a with their corresponding control curves in Fig. 8b, it can be observed that the values of the optimal control follow a sinusoidal-like evolution being aligned with the sinusoidal evolution of users’ interest. The decreasing with time trend in the optimal control values, which cannot be explained by the $\Gamma$ function (Eq. 23, "Optimal control framework for information diffusion"), can be justified by the decreasing values of the adjoint variable $\lambda$ with time as shown in Fig. 8c where time is indicated by the arrow.

**Fig. 9:**
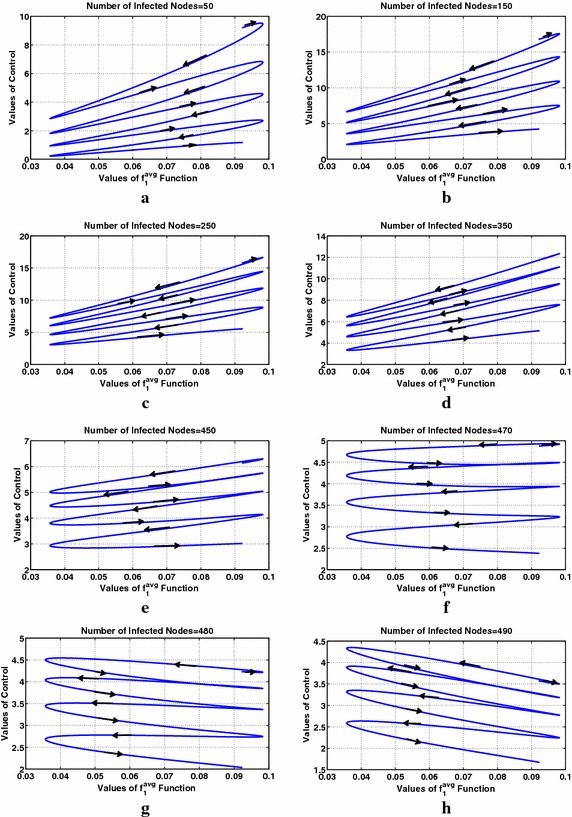
User interest that is periodic with time. Study of the optimal control’s behavior with respect to users’ interest and the number of infected nodes for class 2. The *arrows* denote the time evolution

Finally, Fig. 9 shows the monotonicity of the optimal control with respect to interest for different values of the number of infected nodes for class $m=2$. The applied parameters in these simulations lead to a threshold value $I_{\mathrm{TH}}$ ("Optimal control framework for information diffusion") at most (considering the maximum value of users’ interest) equal to 473 nodes. Thus, it is expected that when $I^{m=2}>473$ nodes the optimal control will be decreasing with interest and the opposite will hold for $I^{m=2}<473$ nodes. This expectation is verified in Fig. 9, where the monotonicity change is performed around $I^{m=2}=470$ nodes, while below 470 the optimal control value increases with interest (Fig. 9a–e) and above this value (Fig. 9g, h) it decreases with interest balancing in this way the cost with the information propagation efficiency as also discussed in "Optimal control framework for information diffusion". Note that in Fig. 9, the ticks on the horizontal axis are not simple interest values, but they are the values of interest over a two-period time interval as shown in Fig. 8a. Therefore, time has also impact on the adjoint variable $\lambda$ affecting the optimal control values leading, e.g., here to decreasing optimal control values with time as it can be observed in all subfigures of Fig. 9. Time evolution is denoted by the arrows in each subfigure of Fig. 9.

### Scenario 2: controlled information diffusion dynamics in the presence of groups with different characteristics

**Fig. 10:**
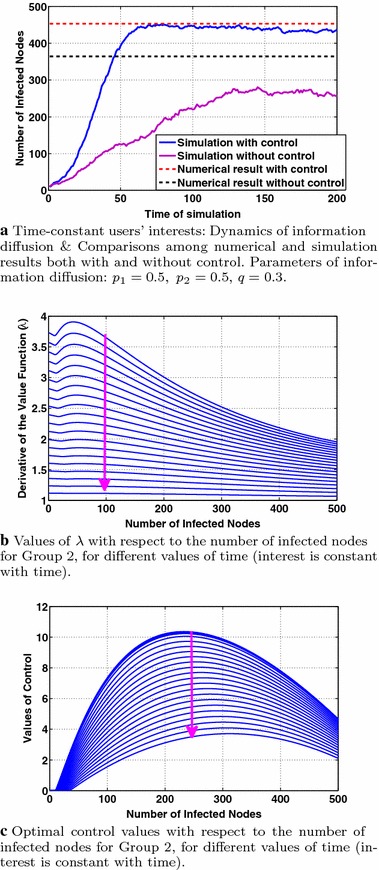
Study of the dynamics of information diffusion and the properties of the optimal control for the case of constant interests. The arrows stand for the time evolution

In this section, we consider constant users’ interests adopting the same parameter values as in "Scenario 2: information diffusion dynamics in the presence of groups with different characteristics". Fig. 10 presents the dynamics of the information diffusion for Group 2 and indicates several properties of the optimal control. Specifically, Fig. 10a depicts the dynamics of information diffusion for Group 2 derived from simulations and computed numerically both cases with and without control (Eqs. 1, 10). As in the case of periodic users’ interests, the introduction of control increases the number of infected nodes. Note that the numerical results of Eq. (10) refer to a time interval of length 2 after convergence (see also the discussion in "Scenario 1: information diffusion dynamics in the case of periodic users’ interests"). As it is shown in Fig. 10a, introducing control leads to a tighter approximation of the simulation results from the numerical ones, compared to the absence of control.

Figure 10c studies the behavior of the optimal control with respect to the number of infected nodes and time for the class $m=2$. As in Fig. 8b, we observe that the optimal control is a concave function of the number of infected nodes (for all times) while $I_{\mathrm{\max }}^m$ remains approximately stable (due to the consideration of constant interests). The optimal control values decrease with time (indicated by the arrow) due to the decreasing values of the adjoint variable $\lambda$ with time as shown in Fig. 10b.

### Scenario 3: controlled information diffusion dynamics in the case of decreasing with time users’ interests

**Fig. 11:**
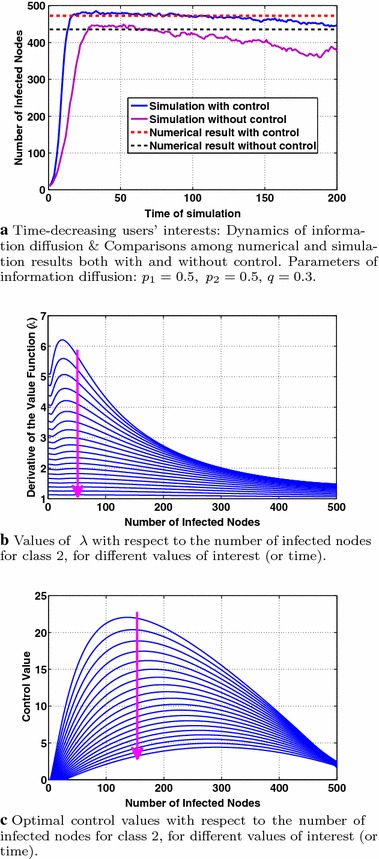
Dynamics of information diffusion and behavior of the optimal control for the case of decreasing with time user interests. The *arrows* stand for the time evolution

In this section, we consider users’ interests that decrease with time using the same parameters/interest functions with "Scenario 3: information diffusion dynamics in the case of increasing vs. decreasing users’ interest". Figure 11 presents the dynamics of the information diffusion for class 2 and indicates several properties of the optimal control. Specifically, Fig. 11a depicts the dynamics of information diffusion for the second class ($m=2$) derived from simulations and computed numerically both cases with and without control (Eqs. 1, 10). We observe that the decrease of users’ interests is reflected to the dynamics of information diffusion, although the later exhibits a much smaller decreasing rate. As in the cases of periodic and constant users’ interests, the introduction of control increases the number of infected nodes. Note that the numerical results of Eq. (10) refer to a time interval of length 2 after convergence (see also the discussion in "Scenario 1: controlled information diffusion dynamics in the case of periodic users’ interests"). As it is shown in Fig. 11a, introducing control leads to a tighter approximation of the simulation results from the numerical ones compared to the absence of control.

Figure 11c studies the behavior of the optimal control with respect to the number of infected nodes and the users’ evolving interest for the class $m=2$. As in Fig. 8b, we observe that the optimal control is a concave function of the number of infected nodes (for all times), achieving its maximum value at a different number of infected nodes, $I_{\max }^m$, for different times (or values of interest) while $I_{\max }^m$ values decrease with interest, as explained in "Scenario 1: controlled information diffusion dynamics in the case of periodic users’ interests". The optimal control values decrease with time (indicated by the arrow) due to the decreasing values of users’ interests and the decrease of the adjoint variable $\lambda$ with time as shown in Fig. 11b.

**Fig. 12:**
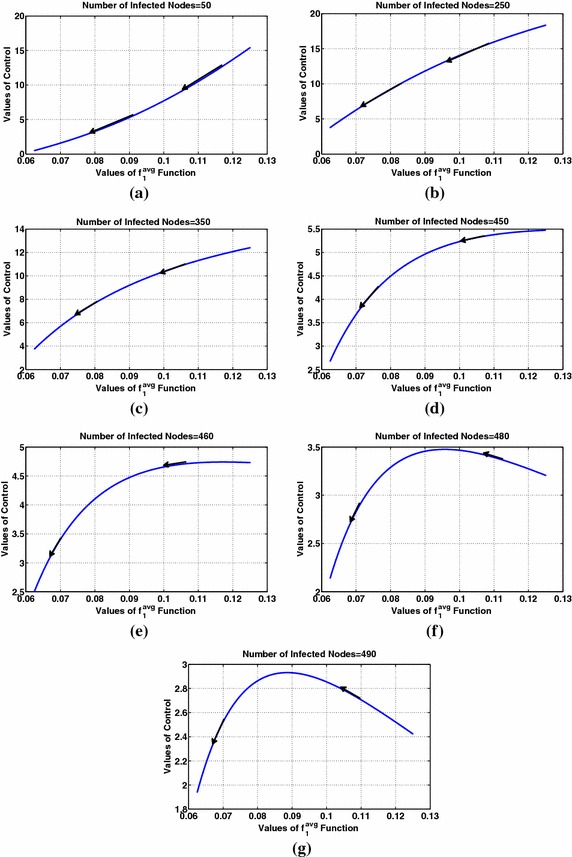
User interest that is decreasing with time: study of the optimal control’s behavior with respect to users’ interest and the number of infected nodes for class 2

Finally, Fig. 12 shows the monotonicity of the optimal control with respect to user interest for different values of the number of infected nodes for class $m=2$. The deployed parameters lead to a threshold value of at most $I_{TH}=475$ ("Optimal control framework for information diffusion") (considering the maximum value of users’ interest). Below $I_{TH}=475$ the optimal control value increases with interest (Fig. 12a–e) and above this value (Fig. 12f, g) it decreases with interest. The dependence of the adjoint variable $\lambda$ on time affects the optimal control values, impacting the monotonicity of the optimal control with respect to user interest and the number of infected nodes, as shown in Fig. 12f, g where the optimal control becomes increasing instead of decreasing (which is expected) with user interest after a specific time. Time evolution is denoted by the arrows in each subfigure of Fig. 12.

## Conclusions

In this paper, we introduced a novel framework for modeling and controlling useful information diffusion in generalized networks that takes into account user interests and their features, i.e., interest periodicity or interest dependence on the topic of the propagated information. The epidemic equations were numerically solved and compared with simulation results for three indicative operational scenarios, yielding significant results on the impact of each associated factor (e.g., topology layer, interest values and time variedness) on diffusion dynamics. Furthermore, optimal controls were obtained and studied over each information class, while simulation and numerical results are provided for the cases where user interest is low and diffusion needs boosting to improve the efficiency of useful information spreading. Interesting behavioral properties of the optimal controls with respect to their dependence on the evolving users’ interests and the number of infected nodes are shown via analysis (on an intuitive basis) and numerical evaluations. Our future work will focus on studying the cases where interest classes may have correlations, and the impact that these correlations may have on the corresponding controls.
